# The Nonlinear Dynamics and Chaos Control of Pricing Games in Group Robot Systems

**DOI:** 10.3390/e27020164

**Published:** 2025-02-04

**Authors:** Chen Wang, Yi Sun, Ying Han, Chao Zhang

**Affiliations:** 1 School of Communication and Information Engineering, Xi’an University of Science and Technology, Xi’an 710054, China; wc2067929277@163.com (C.W.); 23207223143@stu.xust.edu.cn (Y.H.); 23207035002@stu.xust.edu.cn (C.Z.); 2 Xi’an Key Laboratory of Heterogeneous Network Convergence Communication, Xi’an 710054, China

**Keywords:** resource allocation, group robot systems, pricing games, nonlinear dynamics, chaos control

## Abstract

System stability control in resource allocation is a critical issue in group robot systems. Against this backdrop, this study investigates the nonlinear dynamics and chaotic phenomena that arise during pricing games among finitely rational group robots and proposes control strategies to mitigate chaotic behaviors. A system model and a business model for group robots are developed based on market mechanism mapping, and the dynamics of resource allocation are formulated as a second-order discrete nonlinear system using game theory. Numerical simulations reveal that small perturbations in system parameters, such as pricing adjustment speed, product demand coefficients, and resource substitution coefficients, can induce chaotic behaviors. To address these chaotic phenomena, a control method combining state feedback and parameter adjustment is proposed. This approach dynamically tunes the state feedback intensity of the system via a control parameter M, thereby delaying bifurcations and suppressing chaotic behaviors. It ensures that the distribution of system eigenvalues satisfies stability conditions, allowing control over unstable periodic orbits and period-doubling bifurcations. Simulation results demonstrate that the proposed control method effectively delays period-doubling bifurcations and stabilizes unstable periodic orbits in chaotic attractors. The stability of the system’s Nash equilibrium is significantly improved, and the parameter range for equilibrium pricing is expanded. These findings provide essential theoretical foundations and practical guidance for the design and application of group robot systems.

## 1. Introduction

Resource allocation is ubiquitous in various complex systems, whether in human societies, animal populations in nature, or microbial communities. It permeates every aspect of natural complex systems [1,2,3]. The root cause of resource allocation challenges in complex systems lies in the limited availability of environmental resources. Factors such as system uncertainties—variations in resource production capacity, market environmental elements, resource allocation strategies, and the extent to which individuals comprehend environmental information—can all influence resource distribution within a system [4]. The division of labor among multiple individuals serves as a crucial mechanism for achieving resource allocation in complex systems. As an organizational and management strategy, it emphasizes integrating individual behaviors into collective tasks at the group level to enhance efficiency and productivity [5]. For instance, a hallmark of insect societies is the altruistic self-sacrifice exhibited by group members, alongside complex divisions of labor that demonstrate remarkable plasticity in response to ever-changing environmental conditions [6].

Swarm robotics, as a typical class of complex systems, coordinates groups of robots through local rules with the aim of building systems that are more robust and flexible than individual robots, enabling them to better adapt their behaviors to environmental changes [7,8]. The fundamental logic underlying the achievement of collective goals in swarm robotic systems lies in the effective allocation of resources, which is facilitated by the reasonable division of labor among individual robots. Against the backdrop of resource allocation problems, swarm robotic systems exhibit both cooperation and individual competition. Competition arises as “intelligent” individuals within the system employ rational strategies to maximize their own benefits [9,10,11,12,13,14,15]. The competition for limited resources inevitably leads to conflicts of interest among individuals. Game theory provides a powerful mathematical tool and research framework for studying cooperation and competition among multiple individuals [16]. With the rapid development of artificial intelligence technologies, game theory has played an increasingly significant role in emerging interdisciplinary research areas such as social intelligence, machine intelligence, cooperative intelligence, AI safety, and AI ethics. It offers an effective approach to addressing resource allocation problems in swarm robotic systems [17,18].

Chaotic systems are widely present in nature and engineering applications. Their complex dynamic behavior and extreme sensitivity to initial conditions make the study of chaotic characteristics highly significant. Such research facilitates a better understanding and prediction of complex system behaviors, providing effective guidance for their control and application [19]. Similar to economic systems in human societies, chaotic phenomena may arise during the resource allocation games in swarm robotic systems. This is due to the “nonlinear” nature of interactions among robots when resource allocation problems are modeled using market mechanisms, effectively treating the system as a nonlinear dynamical system [20]. Feng discussed the impact of mutual information on the complexity of swarm robot systems and analyzed the chaotic phenomena within the system [9].

Addressing how to avoid and control chaotic states in swarm robotic systems is a pressing challenge in complex systems. As a key branch of nonlinear dynamics, chaos control aims to regulate the dynamic behavior of systems to meet specific application requirements. On one hand, research on chaos control deepens our fundamental understanding of the nature and physics of chaos; on the other hand, its outcomes have been applied across various engineering fields, offering novel solutions and pathways. Examples include the control of cardiac arrhythmias in biomedical applications, the suppression of anomalously high-amplitude noise caused by chaotic motion in electrical circuits, and the prevention of irregular mechanical motions that lead to the premature fatigue and wear of components.

Among the proposed theories and methods for chaos control, two primary strategies are prevalent: (1) parameter adjustment methods, which control chaotic behavior through techniques such as the Ott–Grebogi–Yorke (OGY) method and parametric resonance methods; and (2) state variable feedback methods, including occasional proportional feedback, delayed feedback, proportional pulse feedback, and linear feedback techniques. Practical and effective chaos control methods stem from a deep understanding of the behavior of complex systems, with goals that range from suppressing chaos to maintain system stability to harnessing chaotic properties for innovative functionalities [21,22].

This study focuses on the complex dynamic game processes among individuals in swarm robotic systems, including both the production game processes and the pricing game processes among resource-providing robots. Various influencing factors and interactions in these dynamic game processes lead to the emergence of chaotic characteristics in the nonlinear dynamical system. Based on this, the study analyzes the chaotic properties of the system under multiple parameters during the pricing game phase of resource-providing robots and proposes control strategies to address chaotic behaviors in the system.

The main contributions of this paper are as follows:

1. Based on market mechanism mapping, this study investigates the pricing game phase in resource allocation within swarm robotic systems. A system model and a business model are proposed, linking the purchasing volume of resource-consuming robots with the pricing strategies of resource-providing robots through game theory. The system dynamics are subsequently modeled as a second-order discrete nonlinear system. By parameterizing key influencing factors—such as pricing adjustment speed, demand coefficient, and resource substitution coefficient—and conducting numerical simulations, the results demonstrate that lower pricing adjustment speeds, higher demand coefficients, or lower resource substitution coefficients can enhance the stability of the system’s pricing and revenue. Conversely, slight perturbations in system parameters may trigger chaotic behaviors.

2. To address the chaotic phenomena emerging from competition among individuals, this study proposes control methods based on state feedback and parameter adjustment. By dynamically adjusting the system’s state feedback intensity through the control parameter M, chaotic behaviors are suppressed, and delayed bifurcations are achieved. This ensures that the eigenvalue distribution of the system satisfies stability conditions, thereby enabling the controllability of unstable periodic orbits and period-doubling bifurcations. The proposed methods effectively delay the period-doubling bifurcations in the nonlinear dynamical system and control unstable periodic orbits within chaotic attractors. The stability of the system’s Nash equilibrium is significantly improved, the parameter range for equilibrium pricing is expanded, and the onset of chaotic states in the system is postponed.

The remainder of this paper is organized as follows. Section 2 introduces related work, including recent advances in division of labor and game theory in group robot systems, as well as theoretical foundations and applications of nonlinear system control. Section 3 develops the nonlinear dynamics of group robots with finite rationality during pricing games, modeled as a discrete second-order nonlinear system. Section 4 focuses on parameter-controlled nonlinear dynamics and chaos control, presenting numerical analyses, and proposing state feedback and parameter adjustment strategies. Section 5 concludes the study, identifies current limitations, and suggests future research directions.

## 2. Related Work

### 2.1. System Task Allocation and Game-Theoretic Studies

Research on swarm robotics often focuses on practical applications, addressing specific engineering implementations while rarely delving into the underlying dynamics of system behavior, such as the mechanisms driving system functionality. Studies on task allocation and game theory in swarm robotics primarily address resource allocation issues in complex systems. In human society, resource allocation permeates fields such as economics, healthcare, and transportation. The division of labor, as an important organizational and management strategy, efficiently integrates individual behaviors to enhance collective efficiency. In nature, labor division in insect societies enables highly coordinated group behavior to adapt to dynamic environments. Similarly, swarm robotics systems, as complex multi-agent systems, achieve notable flexibility and robustness in executing complex tasks through rational task allocation and cooperation [23,24].

Research on the division of labor and collaboration in swarm robotics focuses on optimizing the organization and control of multi-robot systems using distributed artificial intelligence and multi-agent system theories. Current studies suggest that these systems complete complex tasks in dynamic environments through individual task allocation and collaboration [25]. Task allocation emphasizes the separation of functions between resource providers and consumers, while collaboration is based on real-time communication and shared information between agents. Recently, collaborative robotics (a subset of swarm robotics) has become a research hotspot due to its potential applications in industrial manufacturing, healthcare, and environmental monitoring. This is especially true for achieving efficient collaboration in coexisting, unstructured environments of human–robot cooperation. Researchers have focused on improving individual decision-making autonomy, optimizing global task allocation strategies, and improving collective intelligent behaviors under multimodal information conditions [26].

In swarm robot systems, the division of labor and collaboration among individual robots directly determines the efficiency of resource allocation and overall system performance. The resource allocation problem in swarm robot systems shares similarities with that in human socioeconomic systems, as both are governed by similar underlying logic. Based on a market mechanism framework, the individual robots within a swarm system can be categorized into two groups: resource-providing robots and resource-consuming robots.

Resource-providing robots are responsible for producing and supplying resources, generating profits by selling these resources. Their role is analogous to that of sellers in economic systems. Conversely, resource-consuming robots purchase resources to meet their own needs and derive utility, akin to the role of buyers in human socioeconomic systems. By studying the interactions and competition among robots, the dynamic characteristics and underlying patterns of the system can be revealed.

Game theory provides essential tools for analyzing competitive and cooperative behaviors in multi-robot systems. Regarding the interactions among robots, models such as production games, price games, and quality games are frequently employed. On the one hand, in interactions between resource-providing robots and resource-consuming robots, game models focus more on supply–demand dynamics. For instance, price games and production games can effectively analyze strategy choices and benefit distributions under both symmetric and asymmetric information scenarios [9,10,11,12,13,14]. On the other hand, in collaborative scenarios between resource-providing and resource-consuming robots, cooperative game-based resource-sharing models are effective in coordinating resource utilization conflicts among multiple robots, maximizing overall system efficiency [27]. Joint revenue distribution mechanisms are further employed to fairly allocate benefits generated from cooperation, enhancing system stability and sustainability [28].

Moreover, with the increasing heterogeneity in robot groups, hybrid game models that combine the characteristics of non-cooperative and cooperative games better describe the complex relationships of simultaneous competition and cooperation among resource-providing robots. Under the premise of collaboration, robots may engage in localized competition based on factors such as price or quality to enhance individual benefits while optimizing resource allocation strategies under overall system constraints [29].

With the development of multi-agent systems, evolutionary game theory has been applied to coordinate control in multi-agent systems. During system evolution, agents do not rely on pre-designed dynamic equations but adjust their behaviors independently to increase their benefits, capturing the long-term interactive behaviors within robot groups [30]. By introducing production games, the hierarchical relationships between leaders and followers can be effectively described, optimizing the decision-making strategies of leaders. Furthermore, in environments with incomplete information, Bayesian game models provide theoretical support for uncertainty strategies among robots, particularly in scenarios involving implicit strategies or hidden resource allocation rules within groups [31].

In summary, game theory offers a rich modeling framework for multi-robot systems, encompassing competition to cooperation, static to dynamic interactions, and complete to incomplete information settings. These theories not only provide theoretical guidance for task allocation, resource scheduling, and behavior optimization in robot systems but also pave the way for building more intelligent and flexible multi-robot systems.

### 2.2. Research on Nonlinear Systems Control with Chaotic Characteristics

In nonlinear dynamical systems, bifurcation is a hallmark phenomenon, signifying the transition between different states of motion. Bifurcation theory unveils the relationships between these states, encompassing phenomena such as period-doubling bifurcations, Hopf bifurcations, and saddle-node bifurcations. It is frequently applied to analyze system stability and evolutionary pathways. For instance, in the Lorenz system, researchers employ phase space analysis to explore bifurcation diagrams, elucidating the connections between different states of motion [32,33]. Chaos, a hallmark of nonlinear dynamical systems, represents an intricate phenomenon where deterministic laws govern nonlinear behavior, forming an underlying order rather than sheer randomness. Chaos bridges the extremes of predictable periodic systems and unpredictable stochastic processes, although it is often overlooked. People tend to attribute irregularity to randomness and disorder. Classical chaotic systems such as the Lorenz and Chen systems are widely used to investigate nonlinear dynamics in fields like meteorology, chemical reactions, and communication engineering [34,35].

Chaotic control aims to steer a chaotic system toward a desired periodic behavior, such as equilibrium, periodic motion, or quasi-periodic motion, by applying minimal control inputs. By eliminating bifurcation and chaotic behaviors in the target system, chaotic control has become a critical component of chaos-related applications [21]. In recent years, controlling nonlinear systems with chaotic characteristics has emerged as a pivotal topic in nonlinear dynamics. Chaos, characterized by extreme sensitivity to initial conditions, complex dynamic behaviors, and aperiodicity, poses significant challenges to system control.

State feedback control has been used to suppress or transform chaotic behaviors into stable states by designing appropriate control laws based on the real-time feedback of system state variables to address chaotic phenomena in systems. Linear control methods have shown that small-scale linear perturbations can significantly mitigate chaotic behavior [36]. Parameter adjustment control, another effective approach, dynamically modifies the system parameters to transition behaviors from chaos to stability or periodicity. For example, the Pecora and Carroll chaotic synchronization method achieves synchronization control between chaotic systems through parameter coupling, with applications in fields such as communication encryption [37,38]. Recently, intelligent control methods that exploit chaotic characteristics have attracted attention. Researchers have explored adaptive chaotic control algorithms by integrating machine learning and nonlinear optimization techniques. For example, Verma et al. used genetic algorithms to optimize control parameters for chaotic systems, enabling rapid convergence control for complex nonlinear systems [39]. Practical applications of chaotic control have also become prominent. In power systems, nonlinearities lead to dynamic instability and power oscillations, complicating control efforts. The researchers proposed adaptive fractional fuzzy sliding mode control to suppress low-frequency oscillations and improve system stability [40,41]. Similarly, in maritime shipping, nonlinear motions of ships caused by wind and waves were addressed using state feedback strategies with time delay combined with stochastic excitation, achieving control over chaotic dynamics [42].

Swarm robotic systems, as nonlinear dynamical systems, exhibit chaotic characteristics. The internal game processes among robots can display complex chaotic behaviors under specific conditions. While research on multi-agent or UAV systems primarily focuses on system stability or dynamic evolution, the exploration of chaotic characteristics remains limited. Conversely, studies on chaos in real-world systems like economic systems are more mature. Modeling task allocation in swarm robotics through market mechanisms enables deeper insights into price games among resource-providing robots. The chaotic behaviors in such games share similarities with those in economic systems, providing valuable inspiration for understanding system complexity.

Inspired by these studies, this paper adopts a market mechanism framework to model interactions among robots in swarm robotics systems. It investigates competitive processes during price games among resource-providing robots, employing phase space analysis to study chaotic phenomena in the game process. By analyzing bifurcation and stability under multi-parameter conditions, the paper proposes chaos control methods to stabilize emergent chaotic phenomena, aiming to achieve a more stable system.

## 3. Model

### 3.1. System Model

Using game theory, the following generalized model for the swarm robotic system is defined. In the swarm robotic system, the number of individual robots is denoted as *n*, and the population set is represented as *Z* = {1, 2, …, *n*}. Each robot i∈Z is defined as follows:

(1) $$Robot_{i}=\left(S_{i},B_{i},C_{i},U_{i}\right)$$

Among:

(I) “$S_{i}$ (State)”: This reflects the current state information of an individual robot, such as the behavior strategy, position, speed, etc. In the context of this study, it refers to the different roles of the robots, such as providing resources or consuming resources.

(II) “$B_{i}$ (Behavior)”: Behavior is defined as the strategy or decision taken by an individual robot. For a dynamic swarm robotic system, the behavior of a robot can be represented by its adjustment of state, such as changes in speed, direction, etc. If an individual robot exhibits multiple behaviors simultaneously, the following definition applies: ${\mathrm{Behavior}}_{i}=\left(b_{i1},b_{i2},\ldots ,b_{im}\right)$. In some specific cases, the behavior and state of an individual robot can be considered together. In this study, it refers to the resource pricing provided by the resource-providing robots and the resource purchase volume of the resource-consuming robots.

(III) “$C_{i}$ (Communication)”: This represents the communication topology between the individual and its neighbors. For example, the communication relationship between individuals and their neighbors can be reflected by defining adjacency. Specifically, the set of neighbors of a robot can be defined to indicate the communication range. In the swarm robotic system, this refers to the communication topology network structure between robot individuals.

(IV) “$U_{i}$ (Utility)”: This refers to the payoff that an individual robot obtains from choosing a particular strategy. This reflects the reward the robot gains from adopting a strategy in the current game environment. Rational individuals adjust their strategies to maximize their benefits.

### 3.2. Business Model

Similar to most self-organizing systems in nature, the resources in the swarm robotic system are also limited, and individual robots have different resource demands due to various reasons. To achieve reasonable resource allocation and maintain system stability, a division of labor approach is typically employed. In this process, there are both competitive and cooperative behaviors among individual robots. The foundation of this study lies in the competitive process between the individual robots in the swarm robotic system.

In the swarm robotic system, the robots have different roles, and the system’s resources are reasonably allocated through market mechanism mapping. The robots are divided into two categories: resource-providing robots and resource-consuming robots. Their interaction rules are as follows:

First, the interactions between the resource-providing robots are limited by the initial pricing and total quantity of resources. Each resource-providing robot makes independent decisions, determining its resource output and pricing.

Second, the interaction between resource-providing robots and resource-consuming robots is also driven by decision-making. Resource-providing robots are the suppliers of resources and set the initial resource output and pricing. Resource-consuming robots demand resources and decide their resource consumption based on the prices set by the resource-providing robots. Furthermore, the resource-consuming robots formulate purchasing strategies based on the prices and other factors, and purchase resources from the resource-providing robots, adjusting the resource pricing to maximize their benefits.

### 3.3. Price Game Model of Swarm Robots with Bounded Rationality

In the swarm robotic system, the interaction process between resource-providing robots is abstracted as a price game. A classical price game model is used to map and improve the system, followed by analysis. Each resource-providing robot is a bounded rational individual, aiming to maximize its profit. Its output depends on the resource purchase quantity of the resource-consuming robots, which is price-dependent and characterized by a demand function. This leads to an objective description of the nonlinear dynamics of the swarm robotic system. The parameters of swarm robot systems are explained in Appendix A Table A1.

#### 3.3.1. Game Composition

In the swarm robotic system, the interaction process between resource-providing robots is abstracted as a price game. Each resource-providing robot is a rational individual, aiming to maximize its profit. There are resource-providing robots N(N≥2) and resource-consuming robots M(M≥2) in the system. Interactions occur based on the communication network topology. By using the method of virtual competitors, the game between multiple resource-providing robots in the system is converted into a two-player game.

Assume that all the resource-providing robots produce the same type of resource and share the resource market in the system. The strategy choice for each resource-providing robot is resource pricing $P_{i}\left(t\right)\;\left(i=1,2\right)$, to maximize its profit. Price decisions are made in discrete periods t=0,1,2,3⋯. The output of the resource-providing robot $V_{i}\;\left(i=1,2\right)$ at time t is denoted as $q_{i}=q_{i}\left(t\right)$, and the cost function of the resource is denoted as $C_{i}\left(q_{i}\right)\;\left(i=1,2\right)$, which can be either linear or nonlinear. For the sake of simplicity, assume the variable cost function takes a linear form:

(2) $$C_{i}\left(q_{i}\right)=c_{i}\;\left(i=1,2\right)$$

Among which, $c_{i}\;\left(i=1,2\right)$ represents two resource-providing robots $V_{i}\;\left(i=1,2\right)$. In emergency scenarios, the demand function of resource-consuming robots for the resources produced by the two resource-providing robots is as follows:

(3) $$\left\{\begin{array}{c} Q_{1}\left(t\right)=a-b_{1}p_{1}\left(t\right)+d_{1}p_{2}\left(t\right)\\ Q_{2}\left(t\right)=a-b_{2}p_{2}\left(t\right)+d_{2}p_{1}\left(t\right) \end{array}\right.$$

At this point, the resource-consuming robot first determines its resource demand based on the initial pricing $P_{i}\left(t\right)\;\left(i=1,2\right)$ given by the resource-providing robots. Then, it specifies the purchase quantity for the resources produced $Q_{i}\left(t\right)\;\left(i=1,2\right)$ by the resource providers. Finally, the resource-providing robots determine their production quantities $q_{i}=q_{i}\left(t\right)$ based on the resource purchase amount of the resource-consuming robots. The production quantity of the $i\;\left(i=1,2\right)$ resource-providing robot at period t is as follows:

(4) $$\left\{\begin{array}{c} q_{1}\left(t\right)=Q_{1}\left(t\right)=a-b_{1}p_{1}\left(t\right)+d_{1}p_{2}\left(t\right)\\ q_{2}\left(t\right)=Q_{2}\left(t\right)=a-b_{2}p_{2}\left(t\right)+d_{2}p_{1}\left(t\right) \end{array}\right.$$

Among which, $a,b_{i}\;\left(i=1,2\right)$ and $d_{i}\;\left(i=1,2\right)$ are positive constants, with a representing the market capacity for the resource product. $d_{i}$ is the substitution coefficient of one resource provider’s product concerning the other resource provider’s product. $b_{i}$ is the demand coefficient of the product $\left(0\leq d_{i}\leq b_{i}\leq 1\right)$. The substitution coefficient is influenced by many factors, as it is assumed that the resources and products considered are homogeneous. Under the conditions of complete information and other variables held constant, as long as the price of the product offered by resource provider robot 1 is lower than that of resource provider robot 2, the product from robot 1 will dominate the market share. In other words, when the product quality is the same, the lower the price of the resource provided by a robot, the stronger its competitive position in the market. Conversely, if the resource provider robot sets the price too high, resource-consuming robots will opt not to purchase from it; if the price is set too low, resource-consuming robots will flock to buy from it. When the substitution coefficients reach their upper limit, it indicates that the products are perfect substitutes; when the substitution coefficients are zero, it indicates that the products are unrelated. In emergency scenarios, group robot systems are isolated from the outside world, and resources are limited. Therefore, the three key elements that constitute the price game model between resource provider robots are as follows:

- Participants in the game: Resource provider robots $V_{i}\;\left(i=1,2\right)$;
- Strategy set: Resource pricing by the resource provider robots $P\left(t\right)$;
- Payoff function: Resource provider robots $V_{i}\;\left(i=1,2\right)$. The payoff function in the period *t* is (t=0,1,2,3⋯).
  (5) $$U_{i}\left(p_{1},p_{2}\right)=\left(p_{i}-c_{i}\right)q_{i}$$
  Which is among the following:
  (6) $$q_{i}\left(t\right)=\left\{\begin{array}{c} q_{1}\left(t\right)=a-b_{1}p_{1}\left(t\right)+d_{1}p_{2}\left(t\right)\\ q_{2}\left(t\right)=a-b_{2}p_{2}\left(t\right)+d_{2}p_{1}\left(t\right) \end{array}\right.$$

#### 3.3.2. Game Model

In the aforementioned game process, resource provider robots determine their resource pricing to maximize their respective payoffs. To achieve payoff maximization, the marginal profit must satisfy the following:

(7) $$\frac{\partial U_{i}\left(p_{i},p_{j}\right)}{\partial p_{i}}=a-2b_{i}p_{i}+b_{i}c_{i}+d_{i}p_{j}=0\;\left(i,j=1,2,i\neq j\right)$$

Since in reality, each resource provider robot does not have complete market information and cannot fully predict future market changes, their decisions are often based on partial information. It is assumed that the decisions of the two resource provider robots are based on a bounded rational adjustment process, which is a local estimation of the marginal profit from the previous period. If a resource provider robot *i* believes that the marginal profit in the current period *t* is positive, it will continue to raise the price in the next period t+1. Conversely, if the marginal profit is negative, it will lower the price. Thus, the dynamic evolution process of t+1 resource pricing by the resource provider robots *i* is as follows:

(8) $$p_{i}\left(t+1\right)=p_{i}\left(t\right)+v_{i}p_{i}\left(t\right)\frac{\partial U_{i}\left(p_{i},p_{j}\right)}{\partial p_{i}}\;i=1,2\left(t=0,1,2...\right)$$

Here, $v_{i}>0\;\left(i=1,2\right)$ represents the speed at which the profit signal received by the resource provider robot *i* adjusts its resource pricing decision. The dynamic pricing decision game model for the two resource provider robots can be expressed as Equation (9), which reflects the nonlinear dynamics of the system during the price game phase. It can be observed that the system is a second-order time-discrete nonlinear system during the price game.

(9) $$\left.\left\{\begin{array}{c} p_{1}\left(t+1\right)=p_{1}\left(t\right)+v_{1}p_{1}\left(t\right)\left(a-2b_{1}p_{1}\left(t\right)+c_{1}b_{1}+d_{1}p_{2}\left(t\right)\right)\\ p_{2}\left(t+1\right)=p_{2}\left(t\right)+v_{2}p_{2}\left(t\right)\left(a-2b_{2}p_{2}\left(t\right)+c_{2}b_{2}+d_{2}p_{1}\left(t\right)\right) \end{array}\right.\right.$$

## 4. Equilibrium Points and Stability Analysis of the Second-Order Discrete Nonlinear System

This section primarily provides a rigorous mathematical proof of the existence of the Nash equilibrium solution for the second-order discrete nonlinear system and determines the parameter range that constrains the Nash equilibrium points. If the parameters exceed this range, the Nash equilibrium points become unstable. Additionally, through numerical simulation analysis, the impact of global parameter variations on the local stability of the Nash equilibrium in the price game between resource provider robots in the nonlinear system is explored.

### 4.1. Nash Equilibrium of the System

By using the virtual competitor method, the game among multiple resource provider robots in the nonlinear dynamical system is transformed into a two-player game. A Nash equilibrium exists in the price game between the two “virtual” resource provider robots in the model, where the payoff of each resource provider robot reaches a stable state, meaning that no robot can achieve a higher payoff by changing its strategy. The proof of the existence of the Nash equilibrium solution is as follows:

The revenue function of the resource-providing robot $V_{i}\;\left(i=1,2\right)$ at time period *t*(t=0,1,2,3⋯) is given by the following: $U_{i}\left(p_{1},p_{2}\right)=\left(p_{i}-c_{i}\right)q_{i}$. The second-order partial derivative with respect to its pricing strategy is as follows:

(10) $$\frac{\partial U_{i}^{2}\left(p_{i},p_{j}\right)}{\partial {p_{i}}^{2}}=-b_{i}+d_{i}\;\left(i=1,2\right)$$

where $b_{i}\;\left(i=1,2\right\}$ and $d_{i}\;\left(i=1,2\right)$ are positive constants, and $0\leq d_{i}\leq b_{i}\leq 1$. It follows that the second-order partial derivative of the revenue function of the resource-providing robot is negative, indicating the existence of a Nash equilibrium in this game model.

In the price game model of the group robot system, let $p_{i}\left(t+1\right)=p_{i}\left(t\right)$ represent the price; then, the following equation holds:

(11) $$\left.\left\{\begin{array}{c} p_{1}\left(t\right)+v_{1}p_{1}\left(t\right)\left(a-2b_{1}p_{1}\left(t\right)+c_{1}b_{1}+d_{1}p_{2}\left(t\right)\right)=p_{1}\left(t\right)\\ p_{2}\left(t\right)+v_{2}p_{2}\left(t\right)\left(a-2b_{2}p_{2}\left(t\right)+c_{2}b_{2}+d_{2}p_{1}\left(t\right)\right)=p_{2}\left(t\right) \end{array}\right.\right.$$

The system has four equilibrium points, which are as follows:

$$\begin{array}{cc} \; & E_{1}=\left(0,0\right),\;E_{2}=\left(\frac{a+b_{1}c_{1}}{2b_{1}},0\right),\;E_{3}=\left(0,\frac{a+b_{2}c_{2}}{2b_{2}}\right),\\ \; & E_{4}=\left(\frac{2ab_{2}+ad_{1}+2b_{1}b_{2}c_{1}+d_{1}b_{2}c_{2}}{4b_{1}b_{2}-d_{1}d_{2}},\frac{2ab_{1}+ad_{2}+2b_{1}b_{2}c_{2}+d_{2}b_{1}c_{1}}{4b_{1}b_{2}-d_{1}d_{2}}\right) \end{array}$$

Since the resource-providing robots in the group robot system are individually rational and aim to maximize their payoffs, from an economic perspective, the equilibrium points $E_{1},E_{2},E_{3}$ are bounded equilibrium points and represent unstable states. In these points, individual robots can improve their payoffs by altering their strategies, so $E_{1},E_{2},E_{3}$ are not Nash equilibria. However, at the equilibrium point $E_{4}$, where $4b_{1}b_{2}-d_{1}d_{2}\neq 0$, a Nash equilibrium is achieved.

The following studies the local stability of these equilibrium points for the nonlinear dynamical system.

At equilibrium points $E_{1},E_{2},E_{3}$, the Jacobian matrix of the system is as follows:

$$J\left(p_{1},p_{2}\right)=\left(\begin{array}{cc} 1+v_{1}\left(a+b_{1}c_{1}+d_{1}p_{2}-4b_{1}p_{1}\right) & d_{1}v_{1}p_{1}\\ d_{2}v_{2}p_{2} & 1+v_{2}\left(a+b_{2}c_{2}+d_{2}p_{1}-4b_{2}p_{2}\right) \end{array}\right)$$

Based on the Jacobian matrix, the two eigenvalues at the bounded equilibrium points $E_{1}=\left(0,0\right)$ are $\lambda_{1}=1+v_{1}\left(a+b_{1}c_{1}\right),\lambda_{2}=1+v_{2}\left(a+b_{2}c_{2}\right)$. From the previous assumptions about the parameters, it follows that $\lambda_{1}>1,\lambda_{2}>1$, indicating that equilibrium points $E_{1}$ are unstable. From an economic perspective $E_{1}=\left(0,0\right)$, this suggests that the resource pricing for two resource-providing robots is zero, which is not possible. Next, we consider the situation at this point $E_{2},E_{3}$. Due to the symmetric structure of the two points, we simplify the analysis by focusing on the stability. Similarly, at the bounded equilibrium points $E_{2}$, the two eigenvalues are as follows:

$$\lambda_{1}=1-v_{1}\left(\alpha +b_{1}c_{1}\right),\;\lambda_{2}=1+v_{2}\left(\alpha +b_{2}c_{2}\right)+v_{2}d_{2}\frac{\alpha +b_{1}c_{1}}{2b_{1}}$$

Since at least one of the two eigenvalues $\lambda_{2}=1+v_{2}\left(a+b_{2}c_{2}\right)+v_{2}d_{2}\frac{a+b_{1}c_{1}}{2b_{1}}>0$, so $E_{2}$ point is an unstable bounded equilibrium point. Similarly, the point $E_{3}$ is also an unstable bounded equilibrium point.

At the Nash equilibrium point $E_{4}$, the Jacobian matrix of the system is as follows:

$$J\left(E_{4}\right)=\left(\begin{array}{cc} 1-2v_{1}b_{1}p_{1}^{★} & d_{1}v_{1}p_{1}^{★}\\ d_{2}v_{2}p_{2}^{★} & 1-2v_{2}b_{2}p_{2}^{★} \end{array}\right)$$

where

$$p_{1}^{★}=\frac{2ab_{2}+ad_{1}+2b_{1}b_{2}c_{1}+d_{1}b_{2}c_{2}}{4b_{1}b_{2}-d_{1}d_{2}},\;p_{2}^{★}=\frac{2ab_{1}+ad_{2}+2b_{1}b_{2}c_{2}+d_{2}b_{1}c_{1}}{4b_{1}b_{2}-d_{1}d_{2}}$$

The characteristic equation is $\lambda^{2}-\mathrm{tr}J\lambda +\det J=0$.

Where trJ represents the trace of the Jacobian matrix and detJ represents the determinant $J(E_{4})$ of the Jacobian matrix.

$$\begin{array}{cc} & \mathrm{Tr}\left(J\right)=2-2v_{1}b_{1}p_{1}^{★}-2v_{2}b_{2}p_{2}^{★}\\ & \mathrm{Det}\left(J\right)=1-2v_{1}b_{1}p_{1}^{★}-2v_{2}b_{2}p_{2}^{★}+\left(4v_{1}v_{2}b_{1}b_{2}-d_{1}d_{2}v_{1}v_{2}\right)p_{1}^{★}p_{2}^{★} \end{array}$$

It follows that $\mathrm{Tr}{\left(J\right)}^{2}-4\mathrm{Det}\left(J\right)=4{\left(v_{1}b_{1}p_{1}^{*}-4v_{2}b_{2}p_{2}^{*}\right)}^{2}+4d_{1}d_{2}v_{1}v_{2}p_{1}^{*}p_{2}^{*}$.

Since $4b_{1}b_{2}-d_{1}d_{2}\neq 0,0\leq d_{i}\leq b_{i}\leq 1$, it follows that $\mathrm{Tr}{\left(J\right)}^{2}-4\mathrm{Det}\left(J\right)\geq 0$ and the eigenvalues of the Nash equilibrium point $E_{4}$ are real numbers. According to the Jury condition, the necessary and sufficient condition for the stability of the Nash equilibrium point $E_{4}$ is as follows:

$$\begin{array}{cc} & \left(1\right)1+trJ+\det J>0;\\ & \left(2\right)1-trJ+\det J>0;\\ & \left(3\right)\left|\det J\right|<1 \end{array}$$

Thus, it follows that

$$\left\{\begin{array}{c} 1-\mathrm{Tr}\left(J\right)+D\mathrm{et}\left(J\right)=\left(4b_{1}b_{2}-d_{1}d_{2}\right)v_{1}v_{2}p_{1}^{*}p_{2}^{*}>0\\ 1+\mathrm{Tr}\left(J\right)+D\mathrm{et}\left(J\right)=\left(4b_{1}b_{2}-d_{1}d_{2}\right)v_{1}v_{2}p_{1}^{*}p_{2}^{*}+4-4v_{1}b_{1}p_{1}^{*}-4v_{2}b_{2}p_{2}^{*}>0\\ \left|\mathrm{Det}(J)\right|=\left|1-2v_{1}b_{1}p_{1}^{*}-2v_{2}b_{2}p_{2}^{*}+\left(4b_{1}b_{2}-d_{1}d_{2}\right)v_{1}v_{2}p_{1}^{*}p_{2}^{*}\right|<1 \end{array}\right.$$

Simplifying, we obtain the following:

(12) $$4v_{1}b_{1}p_{1}^{*}+4v_{2}b_{2}p_{2}^{*}-4<\left(4v_{1}v_{2}b_{1}b_{2}-d_{1}d_{2}v_{1}v_{2}\right)p_{1}^{*}p_{2}^{*}<2v_{1}b_{1}p_{1}^{*}+2v_{2}b_{2}p_{2}^{*}$$

The Nash equilibrium point $E_{4}$ is stable within the parameter range constrained by Equation (12), but if the parameters exceed this range, the Nash equilibrium point $E_{4}$ becomes unstable. Therefore, the stability of the nonlinear dynamical system at the Nash equilibrium point depends on the global parameters of the system, which are influenced by the parameters in Equation (12).

### 4.2. Local Stability of the System

To better describe the group robot system and understand the impact of global parameter changes on the local stability of the Nash equilibrium in the price game of resource provider robots in the nonlinear dynamical system, a numerical simulation analysis of the dynamic evolution process during the price game phase was conducted using MATLAB R2024a. In system (9), the parameter *a* represents the market capacity of the resource environment, which cannot be controlled by the resource provider robots. According to market economics, we know that during the price competition phase in the market, resources approach a state of market saturation, and the expenditures of resource providers tend to stabilize. Therefore, the change in unit variable costs $c_{1},c_{2}$ is minimal. As a result, we set this parameter $a=2,c_{1}=0.2,c_{2}=0.8$ and proceed with the analysis based on the obtained values.

**Figure 1:**
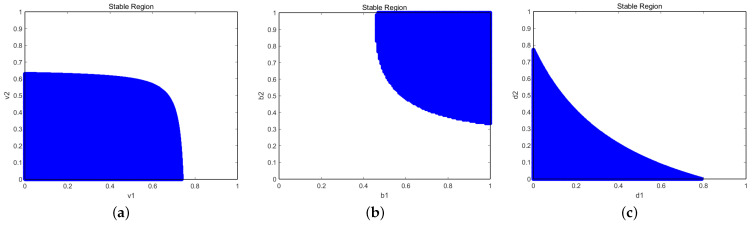
The stable region at the Nash equilibrium. (**a**) The stable region at the Nash equilibrium with $v_{1},v_{2}$. (**b**) The stable region at the Nash equilibrium with $b_{1},b_{2}$. (**c**) The stable region at the Nash equilibrium with $d_{1},d_{2}$.

The group robot system studied in this paper is influenced by each parameter in Equation (12). However, the solution to this equation is relatively difficult, so numerical simulations are used to obtain the simulation region, based on the previous $0\leq d_{i}\leq b_{i}<1$. For rational individual resource-providing robots aiming to maximize personal payoffs, it must hold that $b_{i}\gg d_{i}\;\left(i=1,2\right)$. Using Equation (12), the stable region for the given parameter values $a,c_{1},c_{2},b_{1},b_{2},d_{1},d_{2}$ is determined. The stable region of $v_{1},v_{2}$ is set with the simulation parameters as follows: $a=2,c_{1}=0.2,c_{2}=0.8,b_{1}=0.9,b_{2}=0.9,d_{1}=0.3,d_{2}=0.3$. And the stable region for the given parameter values $a,c_{1},c_{2},v_{1},v_{2},d_{1},d_{2}$ is determined. The stable region of $b_{1},b_{2}$ is set with the simulation parameters as follows: $a=2,c_{1}=0.2,c_{2}=0.8,v_{1}=0.5,v_{2}=0.5,d_{1}=0.3,d_{2}=0.3$. And the stable region for the given parameter values $a,c_{1},c_{2},b_{1},b_{2},v_{1},v_{2}$ is determined. The stable region of $d_{1},d_{2}$ is set with the simulation parameters as follows: $a=2,c_{1}=0.2,c_{2}=0.8,v_{1}=0.5,v_{2}=0.5,b_{1}=0.9,b_{2}=0.9$, as shown in Figure 1.

## 5. Nonlinear Dynamics and Chaos Control of the System Under Parameter Control

### 5.1. Nonlinear Dynamics of the System Under Parameter Control

This section focuses on analyzing the nonlinear dynamics of the system under parameter control through numerical simulation. Specifically, it simulates the bifurcation diagram of resource pricing and payoff for resource provider robots within $v_{1},v_{2}$, the stable region, and also conducts a system dynamics analysis, including the maximum Lyapunov exponent, chaotic attractors, and the basin of attraction. The simulation parameters are set as follows: $a=2,c_{1}=0.2,c_{2}=0.8,b_{1}=0.9,b_{2}=0.9,d_{1}=0.3,d_{2}=0.3$.

#### 5.1.1. Chaotic Characteristics

In the group robot system, the price game model between resource-provider robots is based on the assumption that they possess the same amount of information. Therefore, under fixed conditions for other parameters, the speed of price adjustment $v_{1},v_{2}$ has the same impact on the system’s complexity. Below are the plots of how the resource pricing and payoffs of the resource provider robots in the system change concerning parameter $v_{1}$ variations now that $v_{2}=0.5$, using phase space analysis. The bifurcation diagrams of the price and payoff changes for the resource provider robots in the group robot system concerning parameter $v_{1}$ variations are shown in figures.

**Figure 2:**
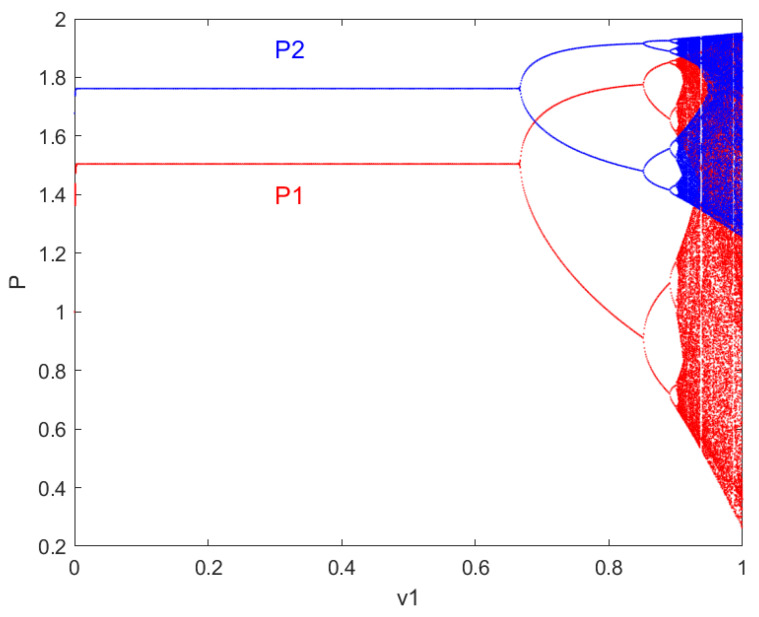
The price $p_{1},p_{2}$ variations of resource-providing robots 1 and 2 with respect to $v_{1}$.

**Figure 3:**
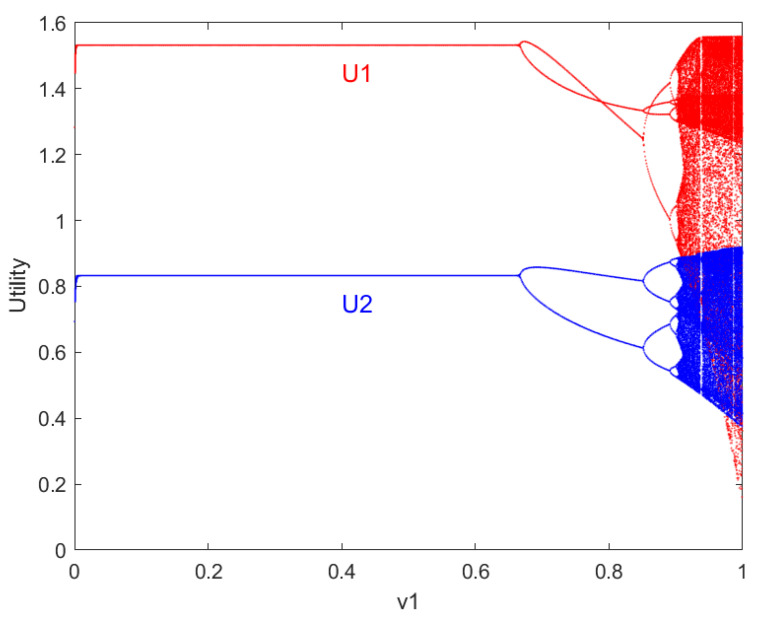
The price $u_{1},u_{2}$ variations of resource-providing robots 1 and 2 with respect to $v_{1}$.

In Figure 2, the bifurcation diagram formed by the red points represents the resource price $p_{1}$ variation of the resource-providing robot 1 changing with $v_{1}$, while the bifurcation diagram formed by the blue points represents the resource price $p_{2}$ variation of the resource-providing robot 2 changing with $v_{2}$. In Figure 3, the bifurcation diagram formed by the red points represents the payoff $u_{1}$ variation of resource-providing robot 1 with respect to $v_{1}$, while the bifurcation diagram formed by the blue points represents the payoff $u_{2}$ variation of resource-providing robot 2 with respect to $v_{1}$.

From the analysis of Figure 2 and Figure 3, it can be observed from the bifurcation diagrams of the price and payoff variations of individual resource-providing robots as the price adjustment speed parameter *v* changes that: the smaller the price adjustment parameter of the resource-providing robots, the more stable their prices and payoffs become, and vice versa. In other words, under fixed conditions for other parameters, when the price adjustment speed $v_{1}$ is too fast, both the prices and payoffs of the robots bifurcate from a single equilibrium point into period-doubling bifurcations, eventually leading to chaotic behavior. Furthermore, as $v_{1}$ gradually increases, the behavior of the group robot system becomes more complex, with even slight increases or decreases in $v_{1}$ causing drastically different state behaviors.

**Figure 4:**
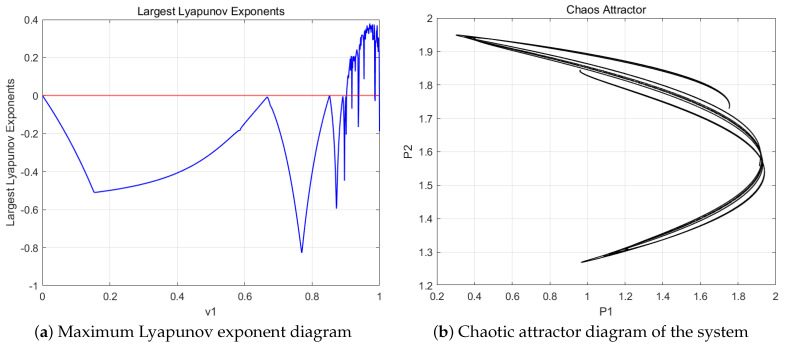
The chaotic state of the system.

Figure 4a shows the maximum Lyapunov exponent of the system with respect to $v_{1}$. The Lyapunov exponent is the average quantity along the system’s trajectory, used to assess the convergence of the phase space trajectory [43]. When the Lyapunov exponent is less than 0, the phase space trajectory converges, and the system is in a stable state. When the Lyapunov exponent is greater than 0, the phase space motion diverges, and the system is in a chaotic state. At this point, $v_{1}=0.5$; when $0\leq v_{1}\leq 0.852$, the Lyapunov exponent is negative, corresponding to a stable periodic state of the system. When $0.852<v_{1}\leq 1$, the Lyapunov exponent is non-negative, meaning there are no stable fixed points or periodic windows within the chaotic zone, and the system enters a chaotic state.

The results indicate that, under certain conditions, the resource pricing behavior of robots $V_{i}\;\left(i=1,2\right)$ will transition from a stable state to a chaotic orbit through period-doubling bifurcations as the price adjustment speed $v_{1}$ increases. The local stability interval $0\leq v_{1}\leq 0.667$ for the Nash equilibrium point $E_{4}$, after which flipping bifurcation occurs at $v_{1}=0.667$. As the adjustment speed $v_{1}$ continues to increase, period-doubling bifurcations occur, eventually leading to higher-order periodic bifurcations and chaos. In other words, starting from a locally stable set of parameters at the Nash equilibrium, increasing the price adjustment speed leads to the loss of stability, eventually resulting in chaotic behavior.

For the chaotic state of the system, mathematical terminology in phase space can consistently describe the actual chaotic phenomena. Although there are no external properties that can be summarized, the inherent patterns of the system can still be characterized. As an indicator of actual chaotic phenomena, the attractor of the system $v_{1}=0.99$ is shown in Figure 4b, which illustrates the chaotic attractor of the model, the bifurcation of periods, and the unstable periodic orbits within the chaotic attractor exhibited by the nonlinear dynamical system.

**Figure 5:**
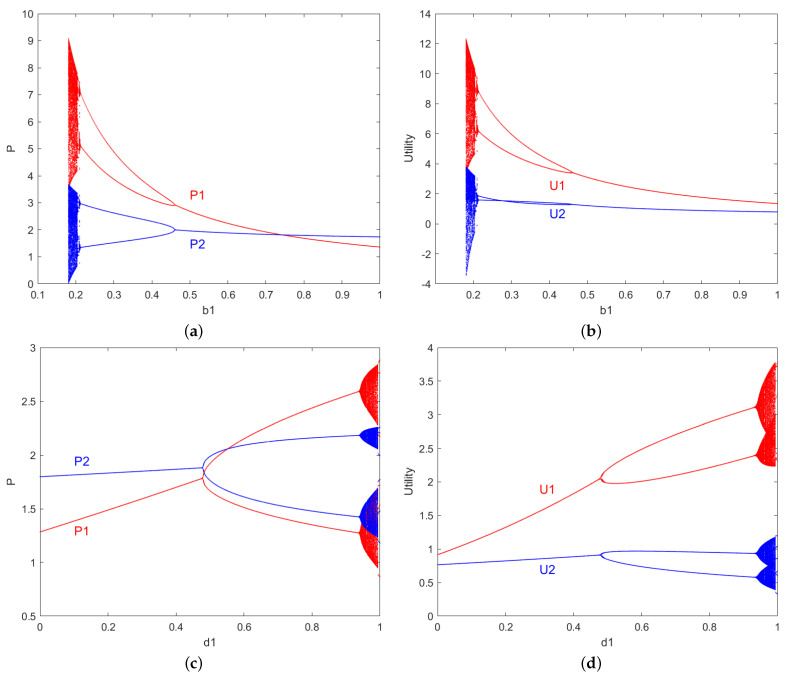
Bifurcation diagram of price and yield influenced by parameters $b_{1},b_{2},d_{1},d_{2}$. (**a**,**c**): The price $p_{1},p_{2}$ variations of resource-providing robots 1 and 2 with respect to $b_{1}$ and $d_{1}$. (**b**,**d**): The price $u_{1},u_{2}$ variations of resource-providing robots 1 and 2 with respect to $b_{1}$ and $d_{1}$.

In the numerical simulation process, we found that the stable region of the Nash equilibrium point $E_{4}$ in the nonlinear dynamical system is influenced not only by the price adjustment speed $v_{1},v_{2}$ but also by other system parameters such as the $b_{1},b_{2},d_{1},d_{2}$ product demand coefficient and the product substitution coefficient, as shown in Figure 5. Figure 5a,b show the bifurcation diagrams for the parameter $b_{1}$. As $b_{1}$ increases, resource pricing and payoffs undergo an inverse-period doubling bifurcation process, where the steady-state flips backward and stabilizes, gradually escaping chaos. The larger the resource demand coefficient $b_{1}$ for the resources produced by the resource-providing robots, the more stable the prices and payoffs become; conversely, the smaller the coefficient, the more unstable the system. Figure 5c,d show the bifurcation diagrams related to the substitution coefficient $d_{1}$. We observe that as the substitution coefficient $d_{1}$ increases, the system gradually enters a two-period state and eventually falls into chaos, exhibiting complex behavior through period bifurcations. The smaller the substitution coefficient $d_{1}$ of the resource-providing robots, the more stable the prices and payoffs; conversely, the larger the substitution coefficient, the more unstable the system. This indicates that disturbances in system parameters lead to the loss of stability, and the second-order discrete nonlinear dynamical system exhibits period-doubling bifurcations. This further demonstrates that the pricing adjustment speed, product demand coefficient, and resource substitution coefficient in this second-order discrete nonlinear system lead to chaotic behavior due to slight perturbations in system parameters.

#### 5.1.2. Global Analysis

Building on existing work, we know that local stability analysis mainly focuses on the neighborhood of the Nash equilibrium point. However, local stability analysis does not include information about the size of different attractor regions or the shape of the basins. To overcome this gap and obtain more insight into the long-term behavior of the attractors in the nonlinear dynamical system of the group robot price game model, we conducted a global analysis in this section. In the actual price game process of group robots, even when other factors are determined, the outcome may not be the same because Equation (9) represents a time-discrete and iterative process. Decision-makers typically use the result from the previous time step as the initial condition for the current step, so the choice of the initial value is crucial. By using a basin of attraction analysis, we examine the system’s convergence after a series of games with a given initial value, which reflects the range of possible initial values for the individual robots.

We use the tool of critical sets to study the structure of the attraction basins for irreversible mappings. The numerical simulation is performed as follows: $a=2,c_{1}=0.2,c_{2}=0.8,b_{1}=0.9,b_{2}=0.9,d_{1}=0.3,d_{2}=0.3,v_{2}=711$.

**Figure 6:**
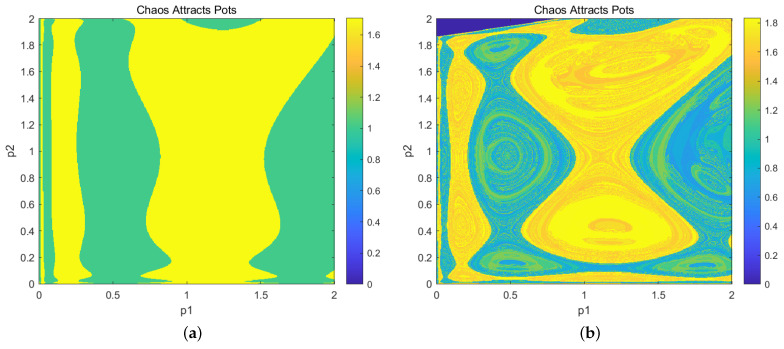
Fixed $a=2,c_{1}=0.2,c_{2}=0.8,b_{1}=0.9,b_{2}=0.9,d_{1}=0.3,d_{2}=0.3$: (**a**) suction basin cycle—2 cycles, $v_{1}=0.707,v_{2}=0.711$; (**b**) attraction basin cycle—4 cycles, $v_{1}=0.852,v_{2}=0.711$.

When set as $v_{1}=0.707$, a stable two-period cycle emerges, and its basin of attraction is shown in Figure 6a. As the $v_{1}$ value increases to 0.852, a stable four-period cycle is formed through a period-doubling bifurcation. Figure 6b illustrates the corresponding basin of attraction. Figure 6 shows the evolution of the attraction basin distribution in a chaotic dynamical system. This evolution not only reflects the complexity of the system’s dynamical characteristics concerning parameter changes but also reveals the profound impact of individual robot resource pricing decisions on the system’s predictability and stability.

As shown in Figure 6, the basin of attraction for the two-period cycle (Figure 6a) exhibits oscillation between two states during the system’s evolution, displaying relatively regular distribution characteristics. Each colored basin region is clear, and the boundaries are well-defined. This indicates that under these parameter conditions, the system’s initial conditions have a clear impact on the final state, and the decision-making process is somewhat predictable. When the parameter value increases to 0.852, the system undergoes a period-doubling bifurcation, evolving from a two-period cycle to a four-period cycle. This means that the system now cycles through four states, which increases its complexity. The corresponding basin distribution (Figure 6b) shows more complex and dispersed boundaries, with more intricate transitional areas between different basins. This indicates increased sensitivity to initial conditions, a decrease in stability, and enhanced chaotic characteristics. Furthermore, the period-doubling bifurcation, as a precursor to chaos, marks the system’s transition from regularity to chaos. In the basin distribution, this transition is reflected in increasingly complex and uncertain boundaries. As the parameter increases further, the basins become more fragmented, eventually leading to a fully chaotic state.

In the group robot system, when the resource pricing adjustment parameters of individual robots are small, the flow of market information and the decision-making environment are relatively stable. The regular distribution of basins indicates that the initial choices of decision-makers have a high degree of predictability regarding market outcomes, providing a stable reference for formulating long-term strategies. However, when individual robots frequently adjust resource pricing, the complexity of the basins is triggered by period-doubling bifurcations. This complexity signifies an increase in market volatility and uncertainty, making it more difficult for decision-makers to predict market trends. The results of the initial decisions may fall into different basins, leading to significant variations in the final state. In the absence of other changes, frequent adjustments in resource pricing by individual robots lower the predictability of market information. The uncertainty of initial decisions becomes substantial, highlighting the need for decision-makers to carefully consider each controllable factor when making choices.

**Figure 7:**
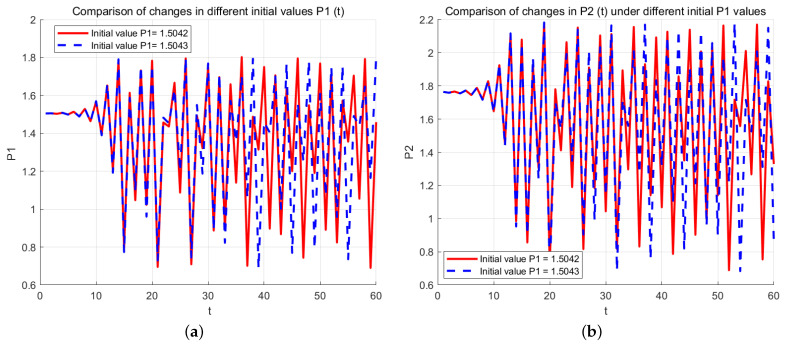
When the system is set as $\alpha =2,c_{1}=0.2,c_{2}=0.8,b_{1}=0.9,b_{2}=0.9,d_{1}=0.3,d_{2}=0.3,v_{1}=0.75,v_{2}=0.75$, the price $P_{1}\left(t\right),P_{2}\left(t\right)$ and it has a sensitive dependence on the initial conditions over the time range [0, 60]. The red solid line corresponds to the initial state $P_{1}\left(0\right)=1.5042,P_{2}\left(0\right)=1.7612$, while the blue dashed line corresponds to the initial condition $P_{1}\left(0\right)=1.5043,P_{2}\left(0\right)=1.7612$. (**a**) Comparison of changes in different initial calues $P_{1}\left(t\right)$. (**b**) Comparison of changes in $P_{2}\left(t\right)$ different initial calues $P_{1}$.

The significance of studying the initial conditions of a nonlinear dynamical system is determined by the inherent randomness of such systems. Figure 7 displays the trajectories of $P_{1}\left(t\right),P_{2}\left(t\right)$, which start from the initial conditions, showing that the differences in the initial states have minimal impact. Both figures include the solid trajectory and the dashed trajectory, corresponding to the initial state $P_{1}\left(0\right)=1.5042,P_{2}\left(0\right)=1.7612$ and $P_{1}\left(0\right)=1.5043,P_{2}\left(0\right)=1.7612$, respectively.

From Figure 7, we observe that although the solid and dashed trajectories, which start with slightly different initial conditions, are difficult to distinguish at the beginning, they quickly diverge as the number of iterations increases. Eventually, the divergence becomes apparent with significant fluctuations. That is, while the results are indistinguishable at the start, after several iterations, the differences between them rapidly increase, illustrating the system’s sensitivity to initial conditions.

### 5.2. State Feedback Parameter Adjustment Chaos Control

Through the above series of numerical simulations, the resource allocation problem in the price game model of the group robot system behaves as a chaotic system. The chaotic phenomena within the system are always triggered by parameter variations. Furthermore, to achieve a stable and reliable group robot system, the instability caused by system parameters must be overcome to facilitate the development of the group robot system’s resource market. The chaos present in the system is undesirable for individual robots and is not a condition the entire nonlinear system wants to maintain. Therefore, the participants in the game strive to find suitable methods to implement effective chaos control.

Luo et al. proposed a method of adding an external variable to achieve state feedback, which has been widely applied in chaos control for nonlinear systems [44,45,46,47]. In this paper, based on this method, we propose a chaos control strategy for the system. Specifically, we apply state variable feedback and parameter adjustment chaos control strategies to Equation (9), resulting in the following Equation (13):

(13) $$\left.\left\{\begin{array}{c} p_{1}\left(t+1\right)=\left(1-M\right)\left[p_{1}\left(t\right)+v_{1}p_{1}\left(t\right)\left(a-2b_{1}p_{1}\left(t\right)+c_{1}b_{1}+d_{1}p_{2}\left(t\right)\right)\right]+Mp_{1}\left(t\right)\\ p_{2}\left(t+1\right)=\left(1-M\right)\left[p_{2}\left(t\right)+v_{2}p_{2}\left(t\right)\left(a-2b_{2}p_{2}\left(t\right)+c_{2}b_{2}+d_{2}p_{1}\left(t\right)\right)\right]+Mp_{2}\left(t\right) \end{array}\right.\right.$$

where M∈(0,1) is the control parameter. Further expansion leads to the following Equation (14):

(14) $$\left.\left\{\begin{array}{c} p_{1}\left(t+1\right)=p_{1}\left(t\right)+\left(1-M\right)v_{1}p_{1}\left(t\right)\left[a-2b_{1}p_{1}\left(t\right)+c_{1}b_{1}+d_{1}p_{2}\left(t\right)\right]\\ p_{2}\left(t+1\right)=p_{2}\left(t\right)+\left(1-M\right)v_{2}p_{2}\left(t\right)\left[a-2b_{2}p_{2}\left(t\right)+c_{2}b_{2}+d_{2}p_{1}\left(t\right)\right] \end{array}\right.\right.$$

By solving for the Nash equilibrium points, we find that Equations (9) and (13) have the same Nash equilibrium, $E^{★}=E_{4}$. These are solved to obtain the following:

(15) $$E^{★}=E_{4}=\left(\frac{2ab_{2}+ad_{1}+2b_{1}b_{2}c_{1}+d_{1}b_{2}c_{2}}{4b_{1}b_{2}-d_{1}d_{2}},\frac{2ab_{1}+ad_{2}+2b_{1}b_{2}c_{2}+d_{2}b_{1}c_{1}}{4b_{1}b_{2}-d_{1}d_{2}}\right)$$

**Figure 8:**
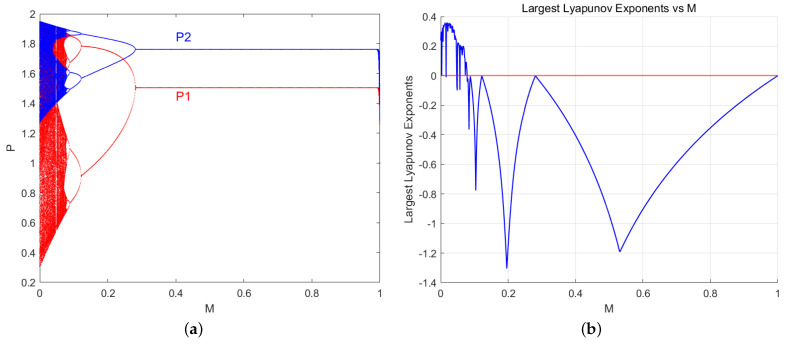
Bifurcation diagram and maximum Lyapunov exponent diagram of price control effect for control parameter *M*. (**a**) Control effect of control parameters. (**b**) The maximum Lyapunov exponent graph of control parameter *M*.

In the chaotic bifurcation diagram section of Figure 2, $v_{1}=0.99$ through setting the other parameters to be consistent with those in Figure 2; we obtain the price bifurcation diagram and the maximum Lyapunov exponent diagram as shown in Figure 8, with the control parameter *M* changes in accordance with Equation (14). In the figure, blue represents resource-providing robot 2, and red represents resource-providing robot 1. Figure 8a shows the control effect of the control parameter (where the price bifurcation diagram undergoes a reverse bifurcation). As the control parameter *M* increases, the system gradually tends to a stable state, $M\in \left(0.266,1\right)$; the pricing stabilizes at the Nash equilibrium. Figure 8b corresponds to the maximum Lyapunov exponent diagram for Figure 8a. In Equation (14) $M\in \left(0,0.083\right)$, when the maximum Lyapunov exponent is greater than zero, the system is in a chaotic state; when the maximum Lyapunov exponent is less than zero, the system is in a periodic state. It can be clearly observed that the controlled price game system at $M\in \left(0,1\right)$ undergoes chaos and bifurcation, and eventually reaches asymptotic stability near the Nash equilibrium point.

**Figure 9:**
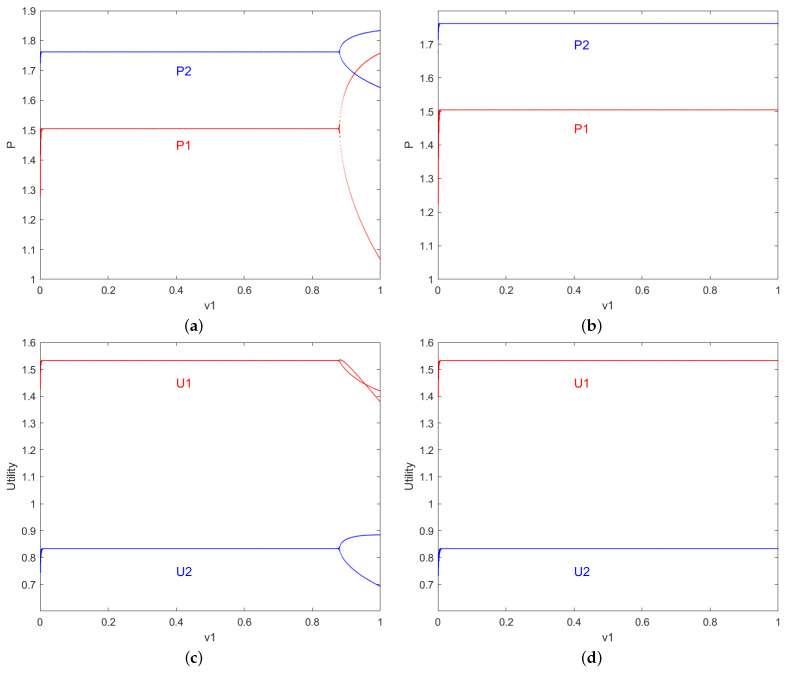
Bifurcation diagrams of resource pricing and profit as the price adjustment speed *v* changes after control are applied. (**a**,**c**): When control parameters M=0.2; The price P,U variations of resource-providing robots 1 and 2 with respect to $v_{1}$. (**b**,**d**): When control parameters M=0.4; The price P,U variations of resource-providing robots 1 and 2 with respect to $v_{1}$.

Figure 9 shows the bifurcation diagrams of resource pricing and profit as the price adjustment speed *v* varies after applying control in Equation (14). In the figure, blue represents resource-providing robot 2, and red represents resource-providing robot 1. Figure 9a is M=0.2 the price bifurcation diagram as resource pricing changes with the adjustment speed *v*. Compared to Figure 2, the first bifurcation point is delayed from $v_{1}=0.667$ to $v_{1}=0.975$. Figure 9b shows the M=0.4 price bifurcation diagram as the system changes with the adjustment speed *v*. Compared to Figure 2 and Figure 9a, during the price game phase of the second-order discrete nonlinear system with the applied control parameter *M*, the game participants adjust control parameters to achieve long-term stable resource pricing.

Figure 9c shows the M=0.2 profit bifurcation diagram as the individual profits change with the adjustment speed *V*. Compared to Figure 3, the first bifurcation point is delayed from $v_{1}=0.664$ to $v_{1}=0.876$. Figure 9d shows the M=0.4 profit bifurcation diagram as the system changes with the adjustment speed *v*. Compared to Figure 3 and Figure 9c, during the price game phase of the second-order discrete nonlinear system with the applied control parameter *M*, the game participants adjust the control parameters to achieve stable long-term profits.

Overall, applying control to the system can delay the instability of the Nash equilibrium point, effectively promoting the stability of the group robot resource market. Based on this controlled second-order discrete nonlinear system mapping, the bifurcation is delayed or eliminated, thereby delaying or preventing the onset of chaotic behavior in the system. The delayed control of the period-doubling bifurcation in the nonlinear dynamical system is effectively achieved, resulting in a significant improvement in the stability of the system’s Nash equilibrium and an expanded range of equilibrium prices.

A primary goal of chaos control is to embed control into the unstable periodic orbits within a chaotic attractor. For this numerical simulation, take the same as above, $a=2,c_{1}=0.2,c_{2}=0.8,b_{1}=0.9,b_{2}=0.9,d_{1}=0.3,d_{2}=0.3$; the Nash equilibrium solution for the price game is given as follows:

$$E^{★}=\left(\frac{2ab_{2}+ad_{1}+2b_{1}b_{2}c_{1}+a_{1}b_{2}c_{2}}{4b_{1}b_{2}-d_{1}d_{2}},\frac{2ab_{1}+ad_{2}+2b_{1}b_{2}c_{2}+d_{2}b_{1}c_{1}}{4b_{1}b_{2}-d_{1}d_{2}}\right)\approx \left(1.51,1.76\right)$$

As shown in Figure 8, the second-order discrete nonlinear group robot system reflection indicates that in the price bifurcation diagram M∈(0.266,1), as the control parameter *M* changes, the pricing stabilizes at the Nash equilibrium solution. M=0.266 at this point, while the unstable two-cycle orbit in the chaotic attractor is stabilized to a one-cycle orbit, and the pricing stabilizes at the Nash equilibrium solution $E^{★}$. Furthermore, the simulation results in the figure show that increasing the value of the control parameter *M* in the second-order discrete nonlinear group robot system reflection causes the system to operate on the $2^{n}$ periodic orbit. The control strategy from Equation (14) allows the system to be controlled to the $2^{n-k}\left(k=1,2,\cdots ,n\right)$ periodic orbit.

## 6. Conclusions

This paper primarily focuses on the stability of the price competition phase among resource-providing robots in a group robot system under the context of resource allocation. It applies game theory, nonlinear dynamics, and chaos theory to the analysis of internal competition among group robots with bounded rationality, specifically studying the system dynamics during the price competition phase. In response to the chaotic phenomena caused by parameter disturbances in nonlinear dynamic systems, we propose a control method based on state feedback and parameter adjustment, which effectively delays period-doubling bifurcations and controls unstable periodic orbits within chaotic attractors, thereby expanding the stable range of the system’s Nash equilibrium.

Based on a market mechanism mapping, this paper presents a business and system model for the group robot system, analyzing relevant operations during the resource allocation process, and developing a reasonable price competition model. By employing game theory, we model the system as a second-order discrete nonlinear system to describe the dynamics of resource allocation in group robots. We further analyze the impact of system parameters, such as price adjustment speed, on system dynamics. The study provides the range of parameter values at which Nash equilibrium is achieved for both parties in the system. Through numerical simulations, we find that small disturbances in parameters such as price adjustment speed, product demand coefficient, and resource substitution coefficient lead to chaotic behavior in the system. This suggests that even small changes within a certain range in parameters like price adjustment speed have a significant impact on the price formation of the entire resource market in the group robot system. Furthermore, to control the chaos emerging in the price competition phase, we propose a state feedback and parameter adjustment control method, which adjusts the system’s state feedback intensity through a dynamic parameter *M*. This method suppresses chaotic behavior while delaying bifurcation, ensuring that the system’s eigenvalue distribution meets stability conditions, thus maintaining the controllability of unstable periodic orbits and period-doubling bifurcations.

Numerical simulation results show that, on one hand, changes in the price adjustment speed, demand coefficient, and substitution coefficient within the system lead to complexities in the price and profit dynamics of resource-providing robots. The lower the price adjustment speed, the higher the demand coefficient, or the lower the substitution coefficient, the more stable the prices and profits of resource-providing robots. Conversely, instability increases when these parameters are adjusted in the opposite direction. On the other hand, the proposed state feedback and parameter adjustment control method is beneficial in delaying period-doubling bifurcations and controlling unstable periodic orbits within chaotic attractors, which significantly improves the stability of the system’s Nash equilibrium, expands the parameter range for equilibrium prices, and delays the onset of chaos.

This study has certain limitations: the goal of each robot in the group is to maximize its profit, but individual decisions must consider both current and long-term profits, which requires a balance between the two objectives. Additionally, by applying the virtual competitor method, the game between multiple resource-providing robots is simplified to a two-player game, which may not accurately reflect the real price competition between robots in a group system, as the game could involve multiple players.

To overcome these limitations, we outline the following plans for future work: The balance between short-term and long-term profits should be considered, and an attempt should be made to model the price competition between resource-providing robots as a multiplayer game, thereby bringing the model closer to real-world group robot systems. Furthermore, based on the characteristics of the game model and the controllability of parameters causing instability in the system, research should investigate different chaos control methods for group robot systems and their impact on the system’s Nash equilibrium points to identify specific chaos control methods that can maintain the system in a stable state over the long term.

## Data Availability

Based on the data privacy policy, if you are interested in the code, you can contact the corresponding author for the original code in this study, and we will provide it to you. However, we do not disclose all of our simulation code here.The raw data supporting the conclusions of this article will be made available by the authors on request.

## Appendix A

**Table A1:** Parameters of Swarm Robot Systems.

Parameters	Description
$P_{i}\left(t\right)\;\left(i=1,2\right)$	Resource provides the pricing of the supply resource of the robot
$E_{i}\;\left(i=1,2,3,4\right)$	The four equilibrium points of system
$q_{i}$	Resources to provide the yield of the robot
$c_{i}$	Total variation cost per unit resource
$Q_{i}\left(t\right)$	Resource purchase amount of resource consumption robots
*a*	The market capacity of the resource products
$d_{i}$	The substitution coefficient of the resource product to the other resource
$b_{i}$	Demand factor of the product
$V_{i}$	Resources provide robots
$U_{i}\left(p_{1},p_{2}\right)$	The utility function of resource-providing robots
$v_{i}$	Resources provide the speed at which the robot adjusts its resource pricing decisions
*M*	controlling parameter
